# *Dmd^mdx^/Large^myd^*: a new mouse model of neuromuscular diseases useful for studying physiopathological mechanisms and testing therapies

**DOI:** 10.1242/dmm.011700

**Published:** 2013-06-20

**Authors:** Poliana C. M. Martins, Danielle Ayub-Guerrieri, Aurea B. Martins-Bach, Paula Onofre-Oliveira, Jackeline M. Malheiros, Alberto Tannus, Paulo L. de Sousa, Pierre G. Carlier, Mariz Vainzof

**Affiliations:** 1 Laboratory of Muscle Proteins and Comparative Histopathology, Human Genome Research Center, Biosciences Institute, University of São Paulo, São Paulo, Brazil; 2 Centro de Imagens e Espectroscopia *in vivo* por Ressonância Magnética – Instituto de Física de São Carlos – Universidade de São Paulo (CIERMag – IFSC – USP), São Carlos, SP, Brazil; 3 Institut de Myologie, AIM-CEA, Paris, France; 4 Université de Strasbourg, CNRS, ICube (UMR 7357), FMTS, Strasbourg, France

## Abstract

Although muscular dystrophies are among the most common human genetic disorders, there are few treatment options available. Animal models have become increasingly important for testing new therapies prior to entering human clinical trials. The *Dmd^mdx^* mouse is the most widely used animal model for Duchenne muscular dystrophy (DMD), presenting the same molecular and protein defect as seen in humans with the disease. However, this mouse is not useful for clinical trials because of its very mild phenotype. The mouse model for congenital myodystrophy type 1D, *Large^myd^*, harbors a mutation in the glycosyltransferase *Large* gene and displays a severe phenotype. To help elucidate the role of the proteins dystrophin and LARGE in the organization of the dystrophin-glycoprotein complex in muscle sarcolemma, we generated double-mutant mice for the dystrophin and LARGE proteins. The new *Dmd^mdx^/Large^myd^* mouse model is viable and shows a severe phenotype that is associated with the lack of dystrophin in muscle. We tested the usefulness of our new mouse model for cell therapy by systemically injecting them with normal murine mesenchymal adipose stem cells (mASCs). We verified that the mASCs were hosted in the dystrophic muscle. The new mouse model has proven to be very useful for the study of several other therapies, because injected cells can be screened both through DNA and protein analysis. Study of its substantial muscle weakness will also be very informative in the evaluation of functional benefits of these therapies.

## INTRODUCTION

Neuromuscular disorders are a heterogeneous group of genetic diseases that cause a progressive loss of motor ability. There are more than 30 recognized genetically defined forms of neuromuscular disorder, and mutations in many genes causing deficiency or loss of function of different important muscle proteins have been reported as their cause. For instance, defects in components of the dystrophin-glycoprotein complex (DGC) are known to cause Duchenne muscular dystrophy (DMD), sarcoglycanopathies and some forms of congenital muscular dystrophy (Ervasti and Campbell, 1993; Yoshida and Ozawa, 1990).

The DGC is an oligomeric complex composed of dystrophin, sarcoglycans, dystroglycans, sarcospan, syntrophins and α-dystrobrevin. It acts as a link between the cytoskeleton of the muscle cell and the extracellular matrix, providing mechanical support to the plasma membrane during myofiber contraction. Beside this structural function, the complex might also play a role in cellular communication (Campbell and Kahl, 1989; Cohn and Campbell, 2000; Rando, 2001). DMD is caused by mutations in the gene that encodes dystrophin (Hoffman et al., 1987), a protein that is linked through its N-terminal domain to actin and through its C-terminal domain to the integral membrane protein β-dystroglycan (β-DG). The peripheral membrane glycoprotein α-dystroglycan (α-DG), a receptor for the heterotrimeric basement membrane protein laminin-2, binds to β-DG and so completes the connection between intracellular proteins and the extracellular matrix (Straub and Campbell, 1997).

Some forms of muscular dystrophy are associated with genes encoding putative or known glycosyltransferases that are responsible for the appropriate glycosylation of α-DG. Therefore, the importance of post-translational modifications of muscle cell proteins for normal muscle function, in particular α-DG, has become increasingly clear.

Analysis of mouse models for neuromuscular diseases has unraveled previously unknown pathogenetic mechanisms for the development of muscular dystrophy. These animals generally present alterations that are frequently observed in humans with the disease and are therefore important tools for genetic, clinic and histopathological studies, and provide important clues for understanding the pathogenesis of these disorders. Animal models are also very valuable for testing potential therapeutic approaches. The *Dmd^mdx^* mouse is a naturally occurring mutant for DMD, with a stop codon in exon 23 of the murine dystrophin gene. These mice have no detectable dystrophin in the muscle, except in rare revertant myofibers (Hoffman et al., 1987; Bulfield et al., 1984; Sicinski et al., 1989). However, *Dmd^mdx^* mice show a mild phenotype, with comparatively moderate muscle pathology, and muscle degeneration is followed by a large amount of regeneration (Dangain and Vrbova, 1984). Therefore, although the *Dmd^mdx^* mouse is a good genetic and biochemical model for DMD, it is not useful for functional evaluation in therapeutic trials.

**TRANSLATIONAL IMPACT**

**Clinical issue**

Although muscular dystrophies are among the most common human genetic disorders, the treatments that are currently available are palliative rather than curative. A notable member of this group of debilitating disorders is Duchenne muscular dystrophy (DMD), which is caused by mutations in the gene that encodes dystrophin, a key component of the dystrophin-glycoprotein complex (DGC) that connects intracellular proteins with the extracellular matrix. Dystroglycanopathies, another common group of muscular dystrophies, are associated with aberrant glycosylation of α-dystroglycan, which is also an important component of the DGC. The underlying mechanisms, however, remain only partially characterized, and there is an increasing need to establish effective animal models for mechanistic studies and drug screening. The *Dmd^mdx^* mouse is the most widely used animal model for DMD; however, these mice do not fully recapitulate the severity of the human disease. The *Large^myd^* mouse, which harbors a mutation in the glycosyltransferase gene *Large* and presents with aberrant glycosylation, faithfully models congenital muscular dystrophy type 1D. To date, these two models have only been studied separately, which has not allowed the interplay between the dystrophin and LARGE proteins to be investigated.

**Results**

To elucidate the roles of the proteins dystrophin and LARGE in the organization of the DGC in the muscle sarcolemma, the authors generated a double-mutant mouse for the two proteins. Their phenotypic characterization of the *Dmd^mdx^/Large^myd^* mouse model showed that the mice are viable, despite displaying a developmental delay and infertility. Furthermore, the mice exhibited progressive muscular weakness and functional defects. Overall, the phenotype of the double mutant was shown to be more severe than that in either of the parental lineages. Using protein immunohistochemical analysis, the authors confirmed that the severe neuromuscular phenotype correlates with a deficiency of dystrophin in muscle cells. The usefulness of the double-mutant mouse model for testing cell therapy was determined by systemically injecting the mice with normal murine adipose stem cells (mASCs). The mASCs were appropriately hosted in the dystrophic muscle, and could be screened both through DNA and protein analyses.

**Implications and future directions**

This study demonstrates for the first time that combining the genetic defects associated with DMD and dystroglycanopathies does not cause embryonic lethality. However, a deficiency in both the *LARGE* and dystrophin genes gives rise to severe muscular weakness, indicating that the loss of dystrophin together with aberrant glycosylation of α-dystroglycan is more deleterious for muscle tissue than either of the defects in isolation. This novel double-mutant mouse model accurately recapitulates the symptoms of human muscular dystrophies and could be used for further studies of the mechanisms involved in DGC destabilization. In addition, the mouse model has potential utility for the study of stem cell replacement therapy, because injected stem cells can be easily traced using DNA and protein analysis. Thus, the model could have important applications for assessing the molecular effects and functional benefits of forthcoming therapies in individuals with different types of muscular dystrophy.

The myodystrophy *Large^myd^* mouse model harbors a mutation in the gene *Large*, which encodes a glycosyltransferase that presumably alters the glycosylation of α-DG. Mutations in the human *LARGE* gene cause the severe form of muscular dystrophy, congenital muscular dystrophy type 1D. Muscle protein analysis in affected individuals shows hypoglycosylation of α-DG and a consequent reduction of numerous ligand components of the extracellular matrix, such as laminin-2 (Grewal et al., 2005). *Large^myd^* mutant mice develop a progressive myopathy, with a maximum lifespan of 39 weeks.

Clinically, these animals show diffuse and progressive muscle weakness, and widely distributed focal lesions in skeletal muscles as early as 16 days of age. Muscle histopathology includes abnormal muscle morphology, such as degeneration, loss of striation, variation in fiber size and the presence of central nuclei. Several targeted mouse models for DGC-associated muscular dystrophy have been generated. Some double mutants using the *Dmd^mdx^* background have also been created, exhibiting more severe phenotypes (Megeney et al., 1996). These observations have instigated the generation of genetically engineered mouse models for DGC-linked muscular dystrophy.

To better understand the interplay between the lack of dystrophin and LARGE glycosyltransferase in muscle function, we bred *Dmd^mdx^* and *Large^myd^* mice, generating double-mutant animals deficient in these two proteins. In addition to the important physiopathological aspects of the effects of these two mutations in muscle formation and function, this model has an important application for testing therapies, integrating functional, molecular and protein studies. The new *Dmd^mdx^/Large^myd^* mouse model has a severe phenotype that is associated with the lack of dystrophin in the muscle. Therefore, it is a good model for testing cell therapies, because injected cells can be tracked both through DNA analysis for the wild-type allele of the *Large* gene, as well as through the study of the presence of normal dystrophin protein expressed by the injected cells. To test this potential, we systemically injected normal murine adipose mesenchymal stem cells (mASCs) into this new dystrophic model, and we verified that the mASCs were hosted in the dystrophic muscle, as detected by DNA analysis, and expressed traces of dystrophin. These results could have important applications for future therapy in patients with different forms of muscular dystrophy.

## RESULTS

### Generation of *Dmd^mdx^/Large^myd^* mice

**Fig. 1.**
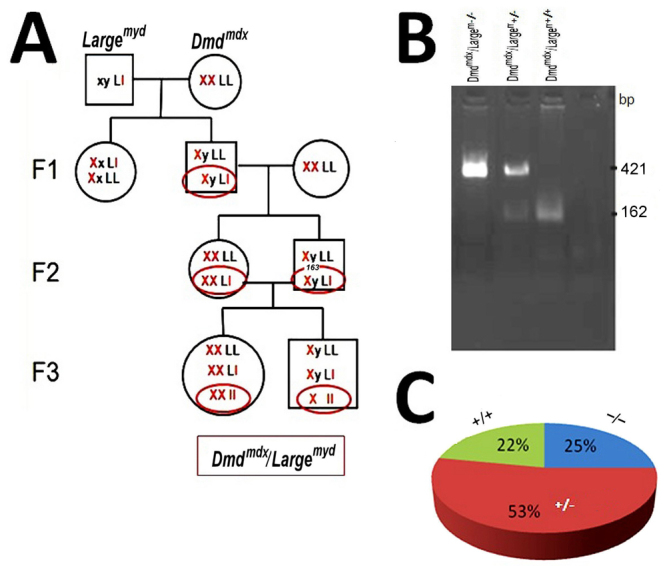
**Generation of the animal model *Dmd^mdx^/Large^myd^***. (A) The breeding program. L, wild-type *Large* allele; l, mutated *Large* allele. (B) The genotyping methodology. The 421 bp band is the mutated allele, and the 162 bp band is the wild-type allele of the *Large* gene. (C) The proportion of each genotype in F3.

The breeding of a male *Large^myd+/−^* with female *Dmd^mdx^* originated 52 animals in F1. The female:male ratio was 47:53, not significantly different from the expected. All male progeny carried the *Dmd^mdx^* mutation in the X chromosome, and were genotyped for the mutant *Large* allele. Sixteen heterozygous *Dmd^mdx^/Large^myd+/−^* male mutants were identified (Fig. 1B) and backcrossed with female *Dmd^mdx^* mice. In F2, we obtained 42 mice, 20 of which were *Dmd^mdx^/Large^myd+/−^* mutants (9 females and 11 males), that were cross-bred for generating the *Dmd^mdx^/Large^myd^* lineage in F3. We obtained a total of 100 newborn mice, with a frequency of each genotype in accordance with mendelian expectations (25 homozygous *Dmd^mdx^/Large^myd−/−^*, 22 wild-type *Dmd^mdx^/Large^myd+/+^* and 53 heterozygote *Dmd^mdx^/Large^myd+/−^*) (Fig. 1C).

### Phenotypic characterization of the *Dmd^mdx^/Large^myd^* mouse

**Fig. 2.**
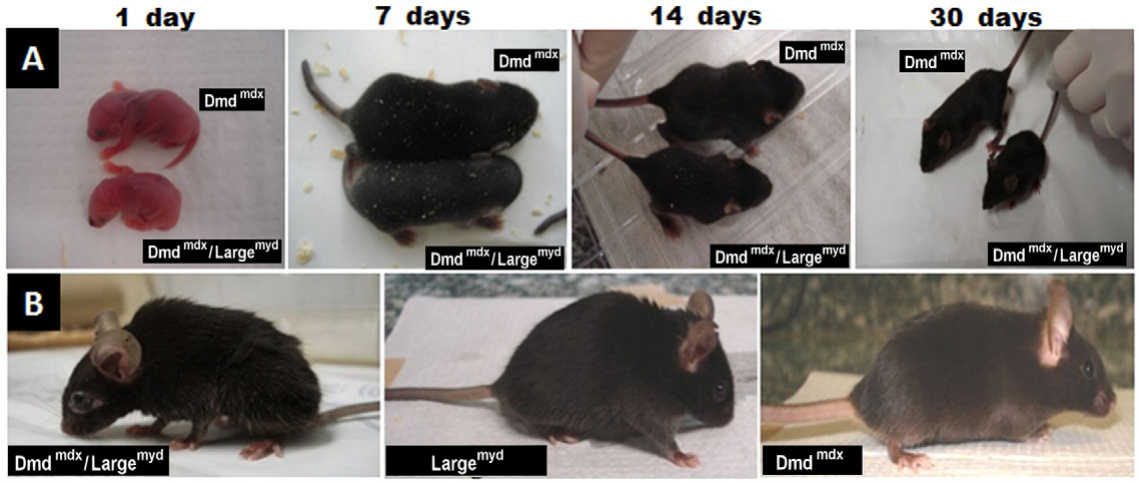
**Phenotypic characterization of the *Dmd^mdx^/Large^myd^* mouse.** (A) Growth and development delay in the *Dmd^mdx^/Large^myd^* double mutant compared with his brother *Dmd^mdx^* at different ages (1, 7, 14 and 30 days). (B) Animals at the age of 120 days.

**Fig. 3.**
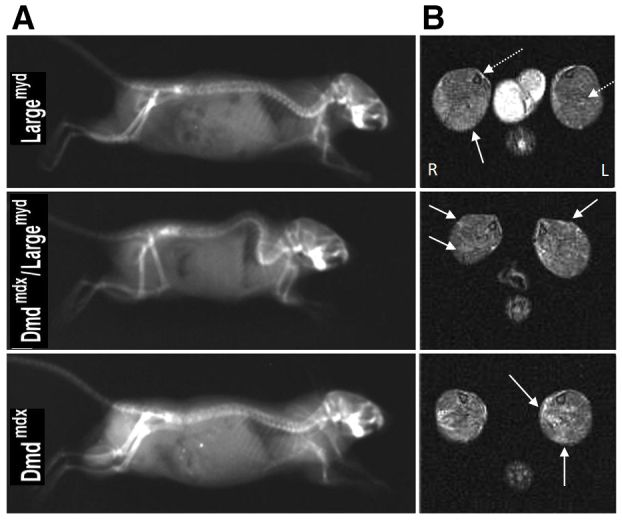
**Image analyses of the *Dmd^mdx^/Large^myc^* model.** (A) Radiography of the spine of the different models (*Large^myd^*, *Dmd^mdx^/Large^myd^* and *Dmd^mdx^*) at the age of 120 days. (B) Axial T2 weighted MRI of 2-month-old *Large^myd^*, *Dmd^mdx^/Large^myd^* and *Dmd^mdx^* mice, at the lower leg level. Solid arrows indicate affected regions; dashed arrows indicate the position of the lower leg bones, tibia and fibula. L, left side; R, right side.

The main features of double-mutant *Dmd^mdx^/Large^myd^* mice were a substantial delay in growth and development, and incapacity of reproduction (Fig. 2). They are smaller at birth and have low neonatal viability: only four among the 25 double mutants survived after the first 24 hours. With the progression of the disease, the mice showed an abnormal phenotype, with decreased body weight, a hunched stance, severe contraction of hind limbs and a stiff walking posture. Compared with the parental *Large^myd^* lineage, the double mutant was weaker and had a reduced maximum lifespan of 24 weeks (in contrast with *Dmd^mdx^*: up to 2 years; and *Large^myd^*: 39 weeks at most) (Lane et al., 1976). Additionally, severe bone alterations were observed with time, and the double mutant developed severe kyphosis. Radiological spinal column analyses at 120 days of age, performed in one mouse from each strain, showed the most severe degree of spinal curvature alteration, when compared with the parental *Dmd^mdx^* and *Large^myd^* lineages at the same age (Fig. 3A).

T2-weighted magnetic resonance images (MRIs) revealed regions of hyper intensities in the calf muscles in the three lineages. Whereas the *Dmd^mdx^* mouse presented a patchy pattern, with several hyper intense points randomly distributed in the calf muscles, the *Large^myd^* mouse presented a hyper intense but homogeneous signal in the posterior compartment, particularly in the soleus muscle. Visually, the double-mutant *Dmd^mdx^/Large^myd^* mouse tended to present a combination of the patterns of the parental lineage: the overall elevation of the muscle signal from the *Large^myd^* model plus the patchy pattern from the *Dmd^mdx^* model (Fig. 3B).

### Functional assessment

**Fig. 4.**
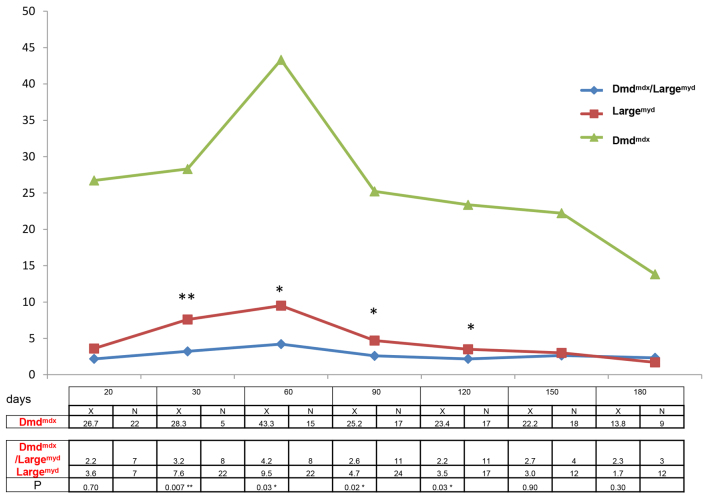
**Functional analysis of the test bar in *Large^myd^*, *Dmd^mdx^/Large^myd^* and *Dmd^mdx^* mice.** *x*-axis, days; *y*-axis, the mean time the mice from each group could hold the bar (seconds). The table below the graph shows the mean time the mice from each group could hold the bar (seconds; X), the number of tested animals (N) and the significance (P) in the Mann-Whitney test, comparing the two weaker strains, *Dmd^mdx^/Large^myd^* versus *Large^myd^* (*P<0.05, **P<0.01).

Functional analysis, comparing *Dmd^mdx^/Large^myd^* mice with the more severely affected parental lineage *Large^myd^* and the milder *Dmd^mdx^* strain, showed that, overall, the double mutant presented a worse performance in the test bar for the front legs (Fig. 4). The differences were statistically significant at the ages of 30 days (*P*=0.007), 60 days (*P*=0.03), 90 days (*P*=0.02) and 120 days (*P*=0.03). After this age, both *Dmd^mdx^/Large^myd^* and *Large^myd^* lineages were similarly impaired.

### Histological and histochemical analyses

**Fig. 5.**
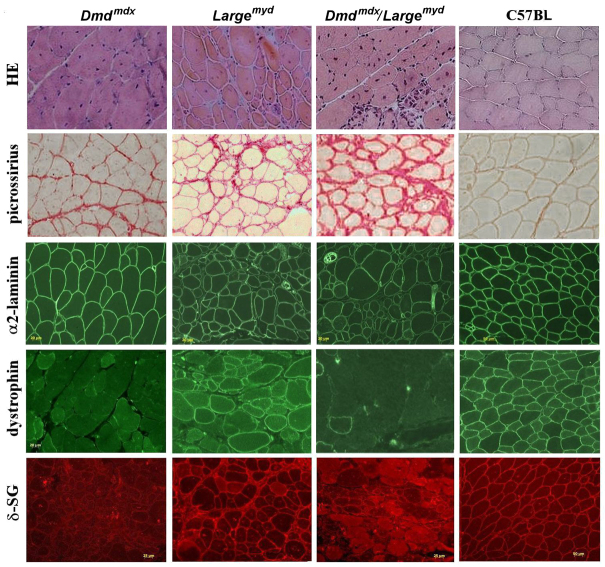
**H&E staining, picrossirius staining for collagens, and immunohistochemical analysis for α2-laminin, dystrophin and δ-SG in normal C57BL, *Dmd^mdx^*, *Large^myd^* and *Dmd^mdx^/Large^myd^* mice aged 180 days.** Magnification 200×.

A comparative histopathological analysis of gastrocnemius muscle of the three dystrophic lineages and the wild-type control is shown in Fig. 5. A similar pattern of muscle degeneration in both *Large^myd^* and *Dmd^mdx^/Large^myd^* was observed in all ages, with variation in fiber size, centrally located nuclei, degenerated fibers, and connective tissue replacement. The *Dmd^mdx^* mice showed the typical pattern of high regeneration, with almost all fibers centronucleated after the age of 3 months. Picrossirius staining showed a crescent amount of connective tissue, from the *Dmd^mdx^*, *Large^myd^* and *Dmd^mdx^/Large^myd^* mice. Protein immunohistochemical analysis revealed a total deficiency of dystrophin in the *Dmd^mdx^* and the double *Dmd^mdx^/Large^myd^* mice, accompanied be secondary deficiency of sarcoglycan proteins, as observed by anti-δ-sarcoglycan (δ-SG) antibody. Interestingly, groups of revertant dystrophin-positive fibers were also observed in the *Dmd^mdx^/Large^myd^* mice, in the same proportion observed routinely in the *Dmd^mdx^* mouse – about six to ten groups of fibers in the whole section.

### mASC therapy in double-mutant *Dmd^mdx^/Large^myd^* mice

**Fig. 6.**
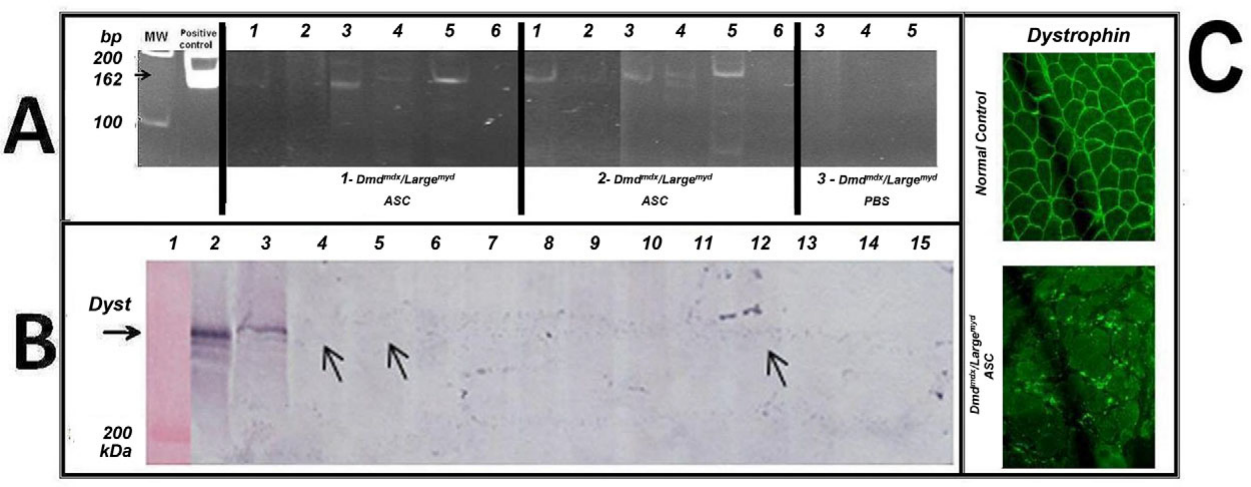
**DNA screening and protein analyses in the *Dmd^mdx^/Large^myd^* model.** (A) DNA screening for the injected cells: PCR for the *Large* normal allele (162 bp) in the injected mice. Animals 1 and 2 were injected with cells, and animal 3 with PBS. Columns: 1, heart; 2, gastrocnemius; 3, stomach; 4, quadriceps; 5, diaphragm; 6, tail. Positive control (162 bp) and molecular weight (MW) are shown. (B) Dystrophin western blot analysis from different muscles from the mASC-injected and PBS-injected animals, reacted with antibody to dystrophin (H300, Santa Cruz Biotechnology, Inc.). Column 1, molecular weight; 2, normal human muscle; 3, normal C57BL muscle. Muscles from *Dmd^mdx^/Large^myd^* mouse 1: column 4, gastrocnemius; 5, quadriceps; 6, diaphragm; 7, heart; 8, stomach. Muscles from *Dmd^mdx^/Large^myd^* mouse 2: 9, gastrocnemius; 10, quadriceps; 11, diaphragm; 12, heart; 13, stomach. Muscles from *Dmd^mdx^/Large^myd^* mouse 3: 14, quadriceps; 15, diaphragm. (C) Dystrophin immunofluorescence analysis in a normal control and in one injected muscle: *Dmd^mdx^/Large^myd^* mouse 2, muscle gastrocnemius.

mASCs were used to assess the utility of the model for future therapies because of their good myogenic potential (Zuk et al., 2002). At 1 week after the last of the three injections, the tissues were collected, and DNA analysis for the normal allele of the *Large* gene identified the presence of the injected cells in the heart, stomach, diaphragm, gastrocnemius muscle and quadriceps muscle (Fig. 6A). Immunofluorescence analysis for dystrophin was negative in the injected muscles (Fig. 6C), whereas western blot analysis identified traces of the 427 kDa dystrophin band in the gastrocnemius, quadriceps and heart muscles (Fig. 6B).

## DISCUSSION

Both the organization and preservation of the proteins of the DGC in the sarcolemma are essential for normal muscle function (Ervasti and Campbell, 1993; Yoshida and Ozawa, 1990, Campbell and Kahl, 1989). Different studies have assessed the interaction of the proteins forming the DGC, both in patients and animal models that mimic human diseases (Vainzof et al., 2008).

The dystrophin protein acts as a link between the cytoskeleton of the cell and the complex contractile machinery within the muscle fiber. Individuals with dystrophin deficiency also have a secondary deficiency in the proteins that make up the DGC (Ohlendieck and Campbell, 1991; Cohn and Campbell, 2000). The LARGE protein is involved in glycosylation pathways and, when altered, a reduction in the molecular weight of α-DG (van Reeuwikj et al., 2007) and a secondary decrease of the protein α-2-laminin are observed. Therefore, both proteins, dystrophin and LARGE, participate in the formation of the DGC. Changes in these proteins destabilize the complex, causing a dystrophic phenotype.

The main goal for the generation of the *Dmd^mdx^/Large^myd^* mouse was to assess the pathological effects of hypoglycosylation in the modulation of the dystrophic phenotype and to estimate the impact caused by the absence of both dystrophin and LARGE proteins in DGC formation.

The double mutant *Dmd^mdx^/Large^myd^* is the first animal model that combines both genetic defects found in DMD and dystroglycanopathies. The first important information is that this animal is viable, with no intrauterine death. Therefore, deficiencies of both dystrophin and LARGE proteins are compatible with life. Accordingly, the double mutant showed greater weakness, when compared with the *Large^myd^* parental lineage, indicating that the loss of dystrophin together with a defect of glycosylation of α-DG is more deleterious for the muscle and worsens the phenotype.

### Functional characteristics of *Dmd^mdx^/Large^myd^* mice

In the present study, we found that, among the 52 animals obtained in F1, the proportion of females and males was similar, and 50% of the *Dmd^mdx^* males also carried the *Large* mutation. This mendelian proportion suggests that there are no deleterious effects for an heterozygous *Large* mutation in the *Dmd^mdx^* phenotype. These males were successfully cross-bred with female *Dmd^mdx^* mice, indicating no reproductive impairment. In F3 we obtained double-mutant animals in the mendelian ratio, demonstrating that the absence of the two proteins did not interfere in the formation of embryos, or in the formation of muscle during development.

However, among the total number of affected animals, only 15% survived after the first 24 hours of life. The cause of death of these animals was not clear, but some signs were observed, such as hypoxia, spasm and abrupt movements of the abdomen, suggesting breathing difficulties in the first hours of life. Respiratory impairment is a common problem in neuromuscular disorders, although dystroglycanopathy generally does not include respiratory distress (Godfrey et al., 2007). Additionally, the *Large^myd^* mouse does not show respiratory problems (Grewal et al., 2005). Nonetheless, further studies will be carried out to assess lung capacity of these animals soon after birth, to better understand the cause of the perinatal death.

The most evident characteristic observed in *Dmd^mdx^/Large^myd^* animals when compared with their *Dmd^mdx^* littermates was their significantly smaller size at birth, which remained smaller throughout their development. By contrast, homozygous *Large^myd^* mice can only be recognized by the smaller size at the age of 12–15 days, when they also present abnormal posturing of their hind limbs (Grewal et al., 2005). This abnormal posture was observed in the double mutants at the same age. *Dmd^mdx^/Large^myd^* animals that reached adulthood lived up to 24 weeks, which is a significantly decreased lifespan when compared with the *Large^myd^* model (39 weeks at most), *Dmd^mdx^* (2 years) and normal controls (2 years). Additionally, the double mutant showed no ability to reproduce, as is also the case for homozygous *Large^myd^* mice. Additional aspects of the phenotype in double mutants included thoracic kyphosis, outward curvature of the spine and hunching of the back, which was evident at 4 weeks and became progressively worse. This alteration can be important for screening the success of therapies, because X-ray measurement can be standardized and compared with check evidence of amelioration of the phenotype. In the homozygous *Large^myd^* mouse, this feature is observed at 6–8 weeks (Grewal and Hewitt, 2002). The observation of more severe features in double mutants suggests that our new *Dmd^mdx^/Large^myd^* mouse model has a more substantial weakness than *Large^myd^* mice.

By contrast, the MRI pattern of the double mutant *Dmd^mdx^/Large^myd^* showed characteristics of both parental lineages. The hyperintensities observed in all three lineages were not related to fat infiltration, because they were observable in the images with and without fat suppression. This indicates that the hyperintensities seen in the muscles of the three lineages were related to inflammation and necrosis. Further studies are in progress to better characterize the muscle involvement in these three mouse lineages using MRI.

### Functional evaluation

Characteristically, young and adult mouse models with muscle weakness, when suspended by the tail, tend to adduct the hind limbs and flex the knee, ankle and toe joints, whereas wild-type mice spread the hind limbs and extend the joints. This aberrant feature was found in *Dmd^mdx^/Large^myd^* mice, being the first observed sign of weakness. To better understand muscle alterations in the *Dmd^mdx^/Large^myd^* mice, we compared its pattern of weakness with the most affected parental lineage, *Large^myd^*. *Dmd^mdx^/Large^myd^* mice presented a statistically significant worse performance in the bar test for the front legs, up to the age of 120 days. After this age, both lineages were equally severely affected. The test was also used for the evaluation of the four legs, with the same poorer performance of the *Dmd^mdx^/Large^myd^* mice compared with *Large^myd^*.

This test evaluates primarily the strength and endurance of these limbs, and is also helpful in evaluating other strength parameters such as balance, ability to use the tail to hold the bar and the use of the rotation of the body to reach the bar (Krämer et al., 1998). Therefore, in general, the new *Dmd^mdx^/Large^myd^* model had a worse performance in the bar for the front legs and four legs, when compared with the *Large^myd^* lineage, demonstrating a greater weakness.

### Histopathological analysis of inbred *Dmd^mdx^, Dmd^mdx^/Large^myd^* and *Large^myd^* mice

For the evaluation of muscle histopathology involvement, we performed morphological and protein analysis by comparing the muscles of double-mutant animals with those from the parental lineages, in different ages. In general, *Dmd^mdx^/Large^myd^* mice showed a similar pattern of degeneration as the *Large^myd^*. However, *Dmd^mdx^/Large^myd^* mice presented a more severe pattern of degeneration at the ages of 21 and 180 days. This included an increase in perimysial and endomysial tissue, a larger variation in fiber size, and an increased proportion of central nuclei and splitted fibers.

The *Dmd^mdx^* mice showed no dystrophin in the muscle, consistent with their genetic defect. *Dmd^mdx^/Large^myd^* mice were also deficient for dystrophin, confirming their genotype. Interestingly, groups of revertant dystrophin-positive fibers were also observed in this new *Dmd^mdx^/Large^myd^* model. The mechanism of revertant fibers has been widely described in the *Dmd^mdx^* mouse, which has a frequency of about 2–3% of positive dystrophin fibers in the muscle. These fibers are also routinely observed in individuals with DMD, as well as in other models for DMD, such as dogs and other mouse lineages with induced mutations in the *Dmd* gene (Danko et al., 1992).

Our results in this double mutant suggest that the introduction of the *Large* mutation does not interfere in the mechanism that produces revertant fibers. Furthermore, in *Dmd^mdx^/Large^myd^* mice we also found the secondary deficiency of sarcoglycan proteins, as observed through the analysis of δ-SG, confirming alterations in the DGC. Alterations in the DGC have not been described in the *Large^myd^* model. Therefore, in our double-mutant model, the lack of dystrophin was probably the most important determinant for the destabilization of the sarcoglycan subcomplex.

### Therapy in *Dmd^mdx^/Large^myd^* mice with adipose stem cells

Stem-cell-based therapies for the repair and regeneration of different tissues, including muscle, is a promising alternative for the treatment of neuromuscular diseases. However, to assess the success of stem cell therapy, we need markers to identify exogenous proteins, indicating the presence of the injected cells in the animal model, as well as functional monitoring to confirm any clinical benefit. In general, animal models for only one mutation do not adequately address these questions in therapeutic preclinical trials. Due to the benign phenotype of *Dmd^mdx^*, it is difficult to monitor clinical benefits from any therapeutic trial. Furthermore, although *Large^myd^* mice are a good functional model, LARGE protein alterations are not easily identifiable in their muscle. Here, as a proof of concept, we preliminarily evaluated the potential of our new model to be used in stem cell transplantation. The homing, retention and differentiation of systemically injected wild-type mASCs was tested in a short time experiment. We observed that the injected cells could reach the affected muscles, because we identified the DNA sequence from the normal allele of the *Large* gene in the different muscles of the injected double-mutant animals. Additionally, we could detect traces of dystrophin in those muscles.

In conclusion, our new *Dmd^mdx^/Large^myd^* model is very useful for stem cell replacement, or any other molecular or pharmacological therapy studies, because we could trace the injected cells/vector/DNA through both DNA and protein analysis. The substantial weakness of these mice will also be very informative to evaluate a functional benefit of these therapies. Additionally, it is important to highlight that any future therapeutic trial must be performed in a perfect window of time to give the appropriate answer. More studies with a larger number of treated animals and longer time frames are necessary to enhance the amount of retained stem cells and to improve the amount of expressed muscle proteins, leading to a better therapeutic effect.

## MATERIALS AND METHODS

### Mice

The two lineages of dystrophic mice, *Dmd^mdx^* and *Large^myd^*, were obtained from our animal facility, in the Human Genome Research Center at University of São Paulo. All lines are available at The Jackson Laboratory. The mice were kept under standard care conditions that minimize stress and optimize breeding. All experiments were approved by the Research Ethics Committee of the Biosciences Institute, University of São Paulo, protocol 098/2009.

Parental lineages were: (1) C57BL/10ScSn-*Dmd^mdx/J^* (MGI: 1856328), also known as *Dmd^mdx^*, a model for DMD. This mouse has a mild phenotype, retains a normal lifespan, and affected males and females are both fertile (Bulfield et al., 1984; Dangain and Vrbova, 1984). (2) B6C3Fe a/a-*Large^myd/J^* (MGI: 1342270), also known as *Large^myd^* (Grewal and Hewitt, 2002) in the C57BL background. The mutation underlying the myodystrophy phenotype has been determined to be an intragenic deletion of exons 5–7, causing a frameshift and a premature stop codon before the first two catalytic domains in the glycosyltransferase LARGE protein. Homozygous mice show abnormal skeletal muscle fiber morphology, decreased body weight, postnatal growth retardation and reduced fertility. The lineage is maintained through the breeding of heterozygous animals.

For histopathological and protein studies, animals were euthanized in a CO_2_ chamber, and tissues were collected and processed for the different analyses. Muscle samples were obtained from gastrocnemius biopsies, frozen in liquid nitrogen immediately after removal, and stored at −70°C until use.

### Production of double-mutants

For F1 production, *Dmd^mdx^* females were mated with *Large^myd+/−^* males. The F1 *Dmd^mdx^/Large^myd+/−^* males were identified by genotyping for the *Large* mutant allele (Browning et al., 2005), and backcrossed to *Dmd^mdx^* females. In F2, both female and male *Dmd^mdx^/Large^myd+/−^* were selected and mated to obtain in F3 the progeny homozygous for the *Large* mutation, i.e. the *Dmd^mdx^/Large^myd^* double mutants (Fig. 1).

### Genotyping for the *Large* mutant and wild-type alleles

The presence of the pathogenic mutation in the studied animals was tested by using multiplex polymerase chain reaction (PCR) analysis for the wild-type and mutated alleles (Grewal et al., 2005). DNA was prepared from mouse-tail tips (0.4–0.6 cm), precipitated with isopropanol and used in a concentration of 50 ng per reaction. For PCR amplification, the described set primers were used: the wild-type 162 bp fragment was amplified with the primers: sense 5′-GGCCGTGGTCCATAAGTTCAA-3′ and antisense 5′-GGCATACGCCTCTGTGAAAAC-3′, and the mutant allele of 421 bp was amplified simultaneously, using the primers sense 5′-ATCTCAGCTCCAAAGGGTGAAG-3′ and antisense 5′-GCCAATGTAAAATGAGGGGAAA-3′.

Both PCR products were amplified in a total volume of 25 μl in the presence of 150 mM dNTPs, 20 pmol primer (each), 1.5 units *Taq* DNA polymerase (Amersham) and 2.5 μl 10× buffer (Amersham). Products were run in 2% agarose gels, stained with ethidium bromide and photographed under UV illumination.

### Magnetic resonance images

The MRI acquisitions were performed in one mouse of each strain (*Dmd^mdx^/Large^myd^*, *Dmd^mdx^*, *Large^myd^* and C57BL) at 2 months of age. The mice were anesthetized with intraperitoneal injection of ketamine/xylazine (2:1, 1.5–2.5 μl/g body weight according to the lineage). The images were acquired in a 2 T/30 cm superconducting magnet (Oxford Instruments 85310HR, Abingdon, UK) interfaced with a Bruker Avance AVIII console (Bruker-Biospin, Inc., Billerica, MA) running PARAVISION 5.0, with a crossed-saddle radiofrequency coil (Papoti, 2006). The sequence parameters were: echo time (TE): 40 ms, repetition time (TR): 1500 ms, 4 averages, slice thickness: 1.5 mm, spatial resolution: 0.176×0.176 mm^2^/pixel.

### Histology and protein analysis

Muscle cryosections were stained with hematoxylin and eosin for histopathological evaluation (Dubowitz and Sewry, 2007). For immunofluorescence analyses, simple or double reactions were done, as previously described (Vainzof et al., 1991). Briefly, four to six cryostat sections were thawed on polylysine-coated slides and air dried for 1 hour. The sections were incubated with primary antibodies overnight at room temperature. The primary antibodies used were: dystrophin-H-300 (Santa Cruz Biotechnology, Inc.), α-2-laminin-4H8-2 (Santa Cruz Biotechnology, Inc.) and δ-sarcoglycan (Nigro et al., 1996). Subsequently, sections were washed three times with PBS, and incubated with secondary antibodies for 1 hour. After three additional washings, the sections were mounted using Vectashield mounting product (Vector Laboratories; London, UK) and analyzed under a Zeiss Axiophot microscope with epifluorescence (Carl Zeiss, Oberkochen, Germany).

The same exposure time was used for registration of all reactions, and image exposure time was standardized using the positive normal control for each reaction/antibody/day. Digital pictures were taken using the Axiovision 4.5 software (Carl Zeiss).

### mASC isolation, expansion and injections

Subcutaneous adipose tissue of adult wild-type C57BL mice was collected, washed with PBS containing 2.5% antibiotics [penicillin and streptomycin (Gibco)] and dissociated with 0.075% collagenase (Sigma) at 37°C for 30 minutes. The collagenase activity was neutralized by DMEM/F12 (Dulbecco’s modified Eagle medium: Nutrient Mixture F-12, Invitrogen) containing 10% FBS (fetal bovine serum; Gibco). The preparation was centrifuged (1200 ***g***, 5 minutes) and the cell pellet was plated in cell culture flasks in DMEM-F12 containing 20% FBS and 2% antibiotics [penicillin and streptomycin (Gibco)]. Subsequently, the cultures were maintained at 37°C in a CO_2_ incubator with 5% humidity. Every 72 hours, the plated cells were washed with 1× PBS, to remove dead cells, and replated when confluent. When the selected cells were predominantly mASCs, they were characterized by flow cytometry, and used for the injection protocol.

The cells were characterized by the expression of membrane proteins that are specific to mASCs (Guava cytometer EasyCyte System, Guava Technologies), and were positive for the markers CD29 (97%), CD105 (86%), CD90 (99%), CD44 (99%), CD13 (85%) and CD31 (80%), and negative for CD34 (4%) and CD45 (10%) (antibodies from BD Biosciences Pharmingen).

The experiment was performed with three dystrophic *Dmd^mdx^/Large^myd^* mice available at the age of 2 months. Two animals received three weekly repeated injections of 10^6^ cells, via the caudal vein, whereas one was injected on the same dates with PBS. All three mice were functionally evaluated before and after starting the injection experiment (in the 4th week), and euthanized for molecular and histological studies.
